# Complete mitochondrial genome of *Tribolium castaneum* (Coleoptera: Tenebrionidae) reared on sauce-flavor Daqu

**DOI:** 10.3389/finsc.2025.1621855

**Published:** 2025-08-29

**Authors:** Xiaomeng Zhang, Rujia Huang, Yubin Chen, Wang Li, Xueqing Zhang, Jianghao Yang, Jun Lv

**Affiliations:** ^1^ Agricultural and Rural Service Center of Moutai Town, Renhuai, China; ^2^ School of Food Engineering, Moutai Institute, Renhuai, Guizhou, China; ^3^ Kweichow Moutai Distillery (Group) Hongyingzi Science & Technology Development Co., Ltd, Renhuai, China

**Keywords:** *Tribolium castaneum*, mitochondrial genome, sauce-flavor Daqu, comparative genomics, genetic variation

## Abstract

The red flour beetle *Tribolium castaneum* (Coleoptera: Tenebrionidae), a cosmopolitan stored-product pest frequently infesting sauce-flavor Daqu (a multi-microbial fermented starter), may experience mitochondrial genome variations under the selective pressure exerted by this enzyme-rich substrate. Here we test whether feeding on sauce-flavor Daqu is associated with mitogenomic differences in *T. castaneum*. We present the complete mitochondrial genome of *T. castaneum* from this environment: a 15,885 bp circular DNA (GenBank PV563855) retaining ancestral insect architecture with 71.81% A+T content and slight positive AT skew. The genome contains 37 functional elements: 22 tRNA genes (all exhibiting atypical cloverleaf structures except trnS1(AGN)), 13 protein-coding genes (PCGs), 2 rRNA genes, and a 1,238 bp A+T-rich control region (82.80% AT). Eleven PCGs initiate with ATN codons, while *cox1* (CTG) and *nad1* (TTG) show divergent initiation. Ten PCGs terminate with TAA/TAG codons. Gene order aligns with basal insect mitogenomes. Comparative analysis with Jiangsu (China) and California (USA) strains revealed conserved structural features, though sequence/assembly discrepancies require further investigation to assess potential pressure-induced mutations. While these differences may reflect adaptations to the enzyme-rich Daqu environment, technical and geographical factors could also contribute; further functional studies are needed to establish causal links.

## 1 Introduction

The red flour beetle, *Tribolium castaneum* (Coleoptera: Tenebrionidae), is a major pest of stored grains and grain products. In sauce-flavor baijiu production regions of China, *T. castaneum* is a predominant pest infesting sauce-flavor Daqu, the critical fermentation starter for this distinctive liquor. Sauce-flavor Daqu, a wheat-based fermentation starter for Chinese baijiu, differs from conventional stored grains by having a porous and thermophilic structure. This substrate supports a diverse microbial community (including *Bacillus* spp., *Aspergillus* fungi, and lactic acid bacteria) and accumulates metabolites like organic acids, and Maillard reaction products. These characteristics may have facilitated the adaptive evolution of *T. castaneum.* Mitochondrial variations could enhance survival in Daqu environments by improving heat tolerance during high-temperature fermentation (60-65 °C) and detoxification of organic acids. Such adaptations may increase infestation persistence, leading to microbial imbalance and starter quality deterioration. Identifying these genomic signatures could inform targeted pest management in baijiu production.

In eukaryotic cells, mitochondria serve as energy-producing organelles primarily through oxidative phosphorylation processes (1). The mitochondrial genome (mtDNA) of insects consists of a circular double-stranded DNA molecule measuring 15,000-18,000 base pairs (2). These maternally inherited genomes employ a variant genetic code for translation (3), maintaining high structural conservation across species. A typical insect mitochondrial genome contains 37 coding elements comprising 13 protein-coding genes, 2 ribosomal RNA (rRNA) genes, and 22 transfer RNA (tRNA) genes, accompanied by a non-coding control region responsible for transcription and replication initiation (2, 4). The non-protein coding components include tRNA genes (identified by their corresponding amino acid designations) and rRNA genes encoding both small (*rrnS*) and large (*rrnL*) mitochondrial ribosome subunits. Protein-coding sequences produce polypeptides essential for electron transport chain complexes: NADH dehydrogenase subunits (complex I, *nad* genes), cytochrome B (complex III, *cob*), cytochrome c oxidase subunits (complex IV, *cox* genes), and ATP synthase components (complex V, *atp* genes).

Prior to this study, two complete *T. castaneum* mitogenomes were available: a 15,881 bp genome from a California laboratory population (NC_003081; Friedrich et al., 2003) (5) and a 15,883 bp genome from a Jiangsu laboratory strain (KM009121; Liu et al., 2016) (6). Both share conserved features including 13 PCGs, 22 tRNAs, and 2 rRNAs. Here, we assemble a third mitogenome from a Guizhou population maintained on sauce-flavor Daqu, representing distinct geographical and dietary conditions. To investigate unresolved questions about substrate-specific variations (e.g., Daqu adaptation) and intercontinental micro-differentiation, we conducted comparative analyses among these three lineages: Guizhou (Daqu-fed), Jiangsu (grain-fed laboratory), and California (grain-fed laboratory).

## 2 Materials and methods

### 2.1 Sample collection

The *T. castaneum* specimens used in this study were collected in 2022 from infested sauce-flavor Daqu in Moutai Town, Guizhou Province, China (27°51′ N, 106°22′ E). These beetles have been maintained on sauce-flavor Daqu in the laboratory since collection. We pooled thirty adult beetles (15 males, 15 females) for analysis and stored them at -80 °C in the Entomology Laboratory of Moutai Institute.

### 2.2 Construction of the genomic library and sequencing

Total genomic DNA was extracted from insects using the TIANamp Genomic DNA Kit (Tiangen, Beijing, China) following the manufacturer’s protocol. DNA concentrations were standardized to 0.3 ng/μL for all samples. Whole genome shotgun (WGS) sequencing was conducted through next-generation sequencing (NGS) technology on the Illumina NovaSeq platform. A library with 400 bp inserts was constructed and sequenced using paired-end methodology at Personalbio (Nanjing, China). Raw sequencing data were transferred to a computer workstation for processing. Initial quality assessment was performed using FastQC v.0.11.9, followed by adapter removal with Trim Galore v.0.6.5. Final data validation was conducted through repeat FastQC analysis.

### 2.3 Genomic assembly

High-quality processed sequencing data were first assembled into Contigs using A5 miseq v20150522 (7), followed by Scaffold construction with SPAdes v3.9.0 (8). Gap filling between contigs was performed through collinearity analysis using Mummer v3.1 (9). Final mitochondrial sequence correction was completed using Pilon v1.18 (10). Assembly accuracy was validated through: (1) BLASTn-confirmed terminal overlaps for circularization, (2) uniform depth distribution averaging 1,580.97× (**Supplementary Figure S1**), and (3) >95% read concordance rate with Q≥30 scores derived from high-quality data (96.97% HQ reads).

### 2.4 Annotation and bioinformatic analysis

The assembled mitochondrial genome was annotated using the MITOS web server (http://mitos.bioinf.uni-leipzig.de) (11). Sequence alignment was performed with SnapGene software using sequences detailed in **Table 1**. The nad3 protein homology model was built with SWISS-MODEL (https://swissmodel.expasy.org/; accessed on 28 July 2025). Bar graphs were drawn using GraphPad Prism 8.0.

**Table 1:** Mitochondrial genomes used in this study.

Species	Population	GenBank accession	Reference
*T. castaneum*	California	NC_003081	(5)
*T. castaneum*	Jiangsu	KM009121	(6)
*T. castaneum*	Moutai	PV563855	This study

## 3 Results

### 3.1 Genome organization and base composition

**Figure 1:**
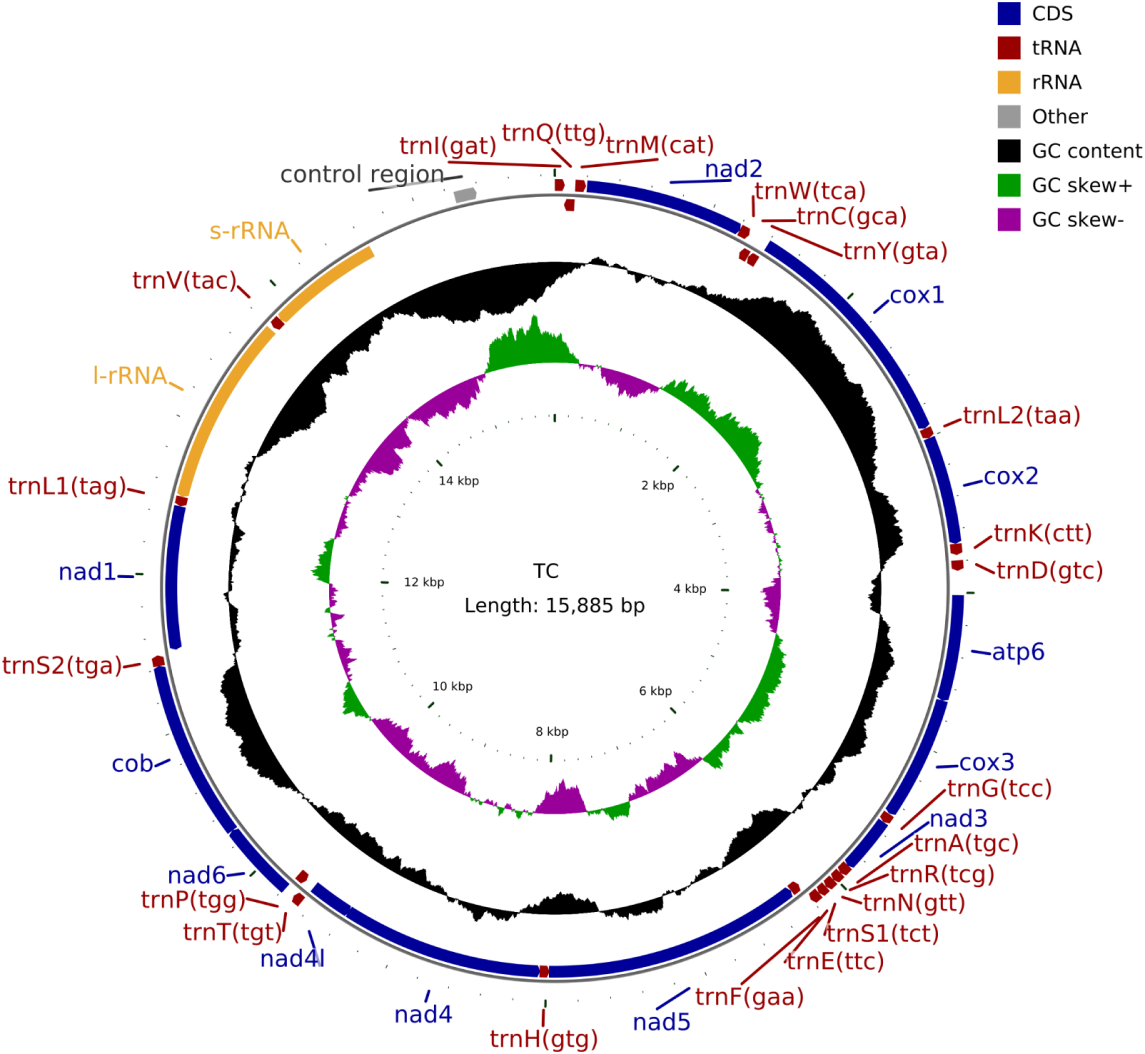
Mitochondrial genome map of *Tribolium castaneum*.

We sequenced the mitochondrial genome of the *T. castaneum* population from Moutai Town, Guizhou, China, reared on Sauce-flavor Daqu. The read sequencing produced 2.9 billion bases (Gb). MiSeq reads corresponding to the mitochondrial genome were separated from nuclear genomic reads (see Methods), with approximately 0.41% of the 19,670,196 total reads representing putative mitochondrial reads. The average sequencing coverage depth was 1580.97×(median = 1580) across the mitochondrial genome (**Supplementary Figure S1**). These reads produced a mitochondrial genome assembly (gMT) of the China isolate of *T. castaneum* with a total length of 15,885 bp (GenBank PV563855), sharing 98.86% identity with the California strain (5) and 98.87% with the Jiangsu strain (6). Features of the mitochondrial genome were annotated to identify 22 tRNA genes, 2 rRNA genes, 13 protein coding genes (7 *nad* subunits, 3 *cox*, 2 *atp*, and 1 *cob*), and a non-coding AT-rich control region (1238 bp) (**Figure 1**). The overall mitogenome exhibited a nucleotide composition of 39.80% A, 9.81% G, 18.38% C, and 32.01% T, with a markedly biased A+T content of 71.81% (**Table 2**). This A+T value slightly exceeds those reported for the California (5) population (71.68%) and Jiangsu (6) laboratory strain (71.00%), suggesting potential lineage-specific genomic adaptations.

**Table 2:** Base composition of different regions of the mitochondrial genome of *T. castaneum*.

Region	A%	C%	G%	T%	A+T%	G+C%	AT_skew	GC_skew
Whole genome	39.8	18.38	9.81	32.01	71.81	28.19	0.109	-0.304
*nd2*	36.42	20	8.76	34.83	71.24	28.76	0.022	-0.391
*cox1*	29.89	22.94	15.92	31.25	61.14	38.86	-0.022	-0.181
*cox2*	37.08	22.34	11.39	29.2	66.28	33.72	0.119	-0.325
*atp8*	45.28	12.58	5.66	36.48	81.76	18.24	0.108	-0.379
*atp6*	34.08	21.28	10.12	34.52	68.6	31.4	-0.007	-0.355
*cox3*	31.93	18.96	14.12	34.99	66.92	33.08	-0.046	-0.146
*nd3*	30.79	20.62	9.89	38.7	69.49	30.51	-0.114	-0.352
*nd5*	26.09	8.41	18.51	46.99	73.09	26.91	-0.286	0.375
*nd4*	25.07	8.98	18.64	47.31	72.38	27.62	-0.307	0.35
*nd4l*	27.08	7.99	17.01	47.92	75	25	-0.278	0.361
*nd6*	34.55	19.8	7.47	38.18	72.73	27.27	-0.05	-0.452
*cob*	31.84	22.89	12.02	33.25	65.09	34.91	-0.022	-0.312
*nd1*	24.82	9.67	17.03	48.48	73.29	26.71	-0.323	0.276
*rrnL*	43.46	16.57	7.38	32.59	76.05	23.95	0.143	-0.384
*rrnS*	34.48	7	17.04	41.48	75.96	24.04	-0.092	0.418
Control region	42.14	4.29	4.29	49.29	91.43	8.57	-0.078	0

### 3.2 PCGs and codon usage

**Figure 2:**
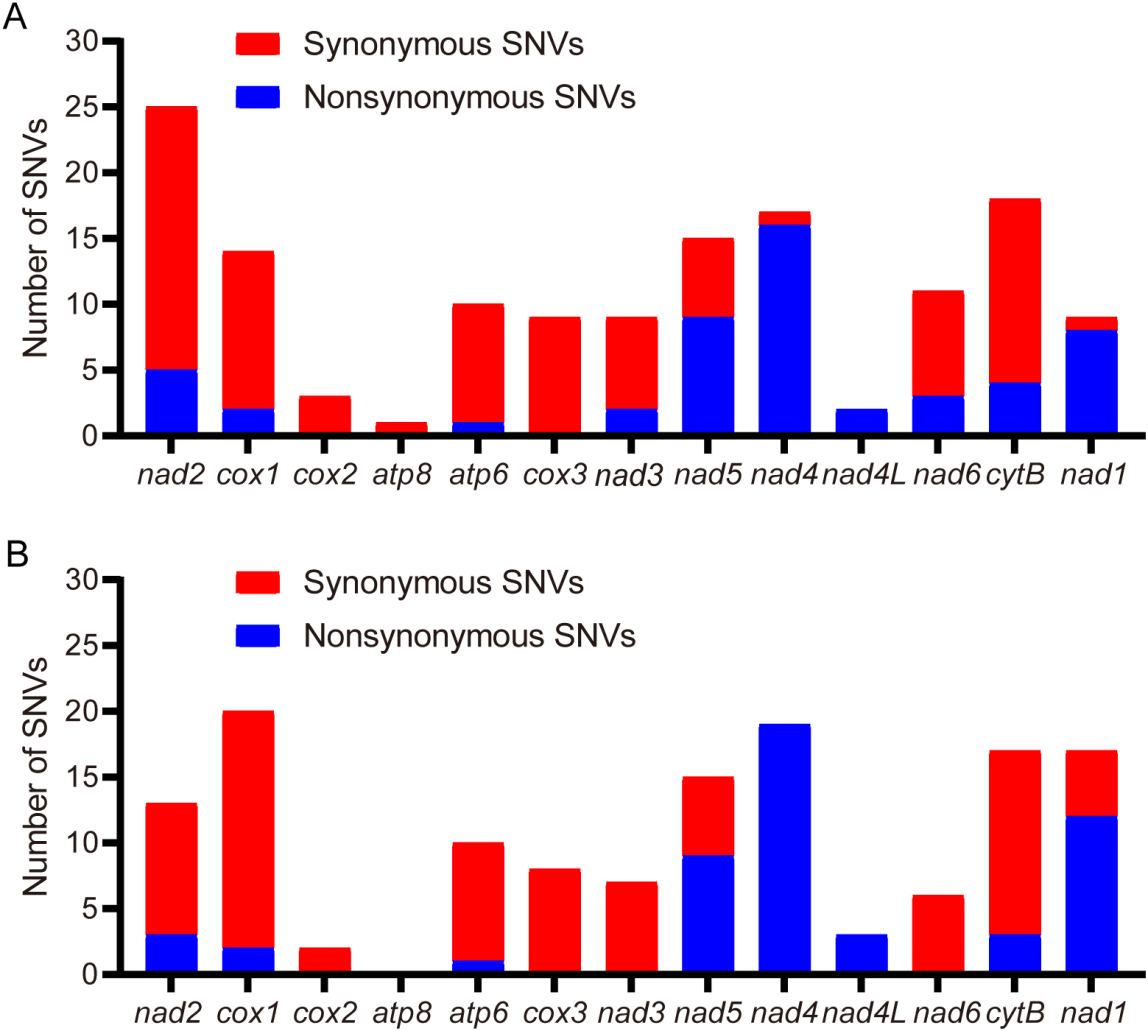
Distribution of single-nucleotide variants (SNVs) in protein-coding genes (PCGs) across *T. castaneum* populations. **(A)** SNV density versus Jiangsu strain (KM009121). **(B)** SNV density versus California strain (NC_003081).

In PCGs, four (*nad4*, *nad4l*, *nad5*, *nad1*) of the 13 PCGs were coded on the N-strand, with the other nine genes (*cox1*, *cox2*, *cox3*, *atp8*, *atp6*, *nad2*, *nad3*, *nad6*, and *cob*) were coded on the C-strand. Among the 13 PCGs, the longest was the *nad5* gene (1,713 bp) and the shortest was the *atp8* gene (159 bp). The start codons of *cox1* (CTG) and *nad1* (TTG) in this study exhibit striking contrast with those of the Jiangsu and California populations (5, 6). Specifically, the Jiangsu population demonstrates AAT for *cox1* and ATT for *nad1*, while the California population (5) utilizes AAA and ATA as start codons for *cox1* and *nad1*, respectively (**Table 3**). Notably, the remaining 11 PCGs uniformly employ ATN start codons across all analyzed populations. Among the 13 PCGs, ten terminated with standard stop codons (TAA/TAG), while *nad5* used ATT, and the remaining two ended with incomplete stops (single T) (**Table 3**). It is generally accepted that incomplete codon structures signal a halt of protein translation in insects and other invertebrates (12). With the exception of *atp8*, *cox2*, and *cox3*, all single-nucleotide variants (SNVs) resulted in nonsynonymous mutations (**Figure 2**). Notably, higher frequencies of nonsynonymous mutations were observed in *nad5*, *nad4*, *nad4l*, and *nad1* (**Figure 2**).

**Table 3:** Summary of the mitogenome of *T. castaneum*.

Feature	Strand	Position(start-end)	Length(bp)	Initiation_codon	Stop_codon	Anticodon	Intergenic_nucleotide
*trnI*	N	1-63	63			GAT	-3
*trnQ*	C	61-129	69			TTG	-1
*trnM*	N	129-196	68			CAT	6
*nd2*	N	203-1,207	1,005	ATA	TAA		-2
*trnW*	N	1,206-1,272	67			TCA	-1
*trnC*	C	1,272-1,332	61			GCA	2
*trnY*	C	1,335-1,398	64			GTA	4
*cox1*	N	1,403-2,941	1,539	CTG	TAA		1
*trnL2*	N	2,943-3,007	65			TAA	
*cox2*	N	3,008-3,692	685	ATA	T(AA)		
*trnK*	N	3,693-3,763	71			CTT	33
*trnD*	N	3,797-3,862	66			GTC	
*atp8*	N	3,863-4,021	159	ATT	TAG		-7
*atp6*	N	4,015-4,686	672	ATG	TAA		-1
*cox3*	N	4,686-5,471	786	ATG	TAA		2
*trnG*	N	5,474-5,535	62			TCC	
*nd3*	N	5,536-5,889	354	ATT	TAG		-2
*trnA*	N	5,888-5,954	67			TGC	-1
*trnR*	N	5,954-6,016	63			TCG	-1
*trnN*	N	6,016-6,079	64			GTT	
*trnS1*	N	6,080-6,138	59			TCT	
*trnE*	N	6,139-6,204	66			TTC	-2
*trnF*	C	6,203-6,267	65			GAA	1
*nd5*	C	6,269-7,981	1,713	ATA	ATT		-3
*trnH*	C	7,979-8,043	65			GTG	-3
*nd4*	C	8,041-9,376	1,336	ATG	T(AA)		-7
*nd4l*	C	9,370-9,657	288	ATG	TAA		2
*trnT*	N	9,660-9,722	63			TGT	
*trnP*	C	9,723-9,788	66			TGG	2
*nd6*	N	9,791-10,285	495	ATC	TAA		-1
*cob*	N	10,285-11,424	1,140	ATG	TAA		-2
*trnS2*	N	11,423-11,490	68			TGA	17
*nd1*	C	11,508-12,458	951	TTG	TAG		
*trnL1*	C	12,459-12,522	64			TAG	
*rrnL*	C	12,523-13,783	1,261				20
*trnV*	C	13,804-13,872	69			TAC	
*rrnS*	C	13,873-14,629	757				623
Control region	N	14,648-15,885	1238				–

**Figure 3:**
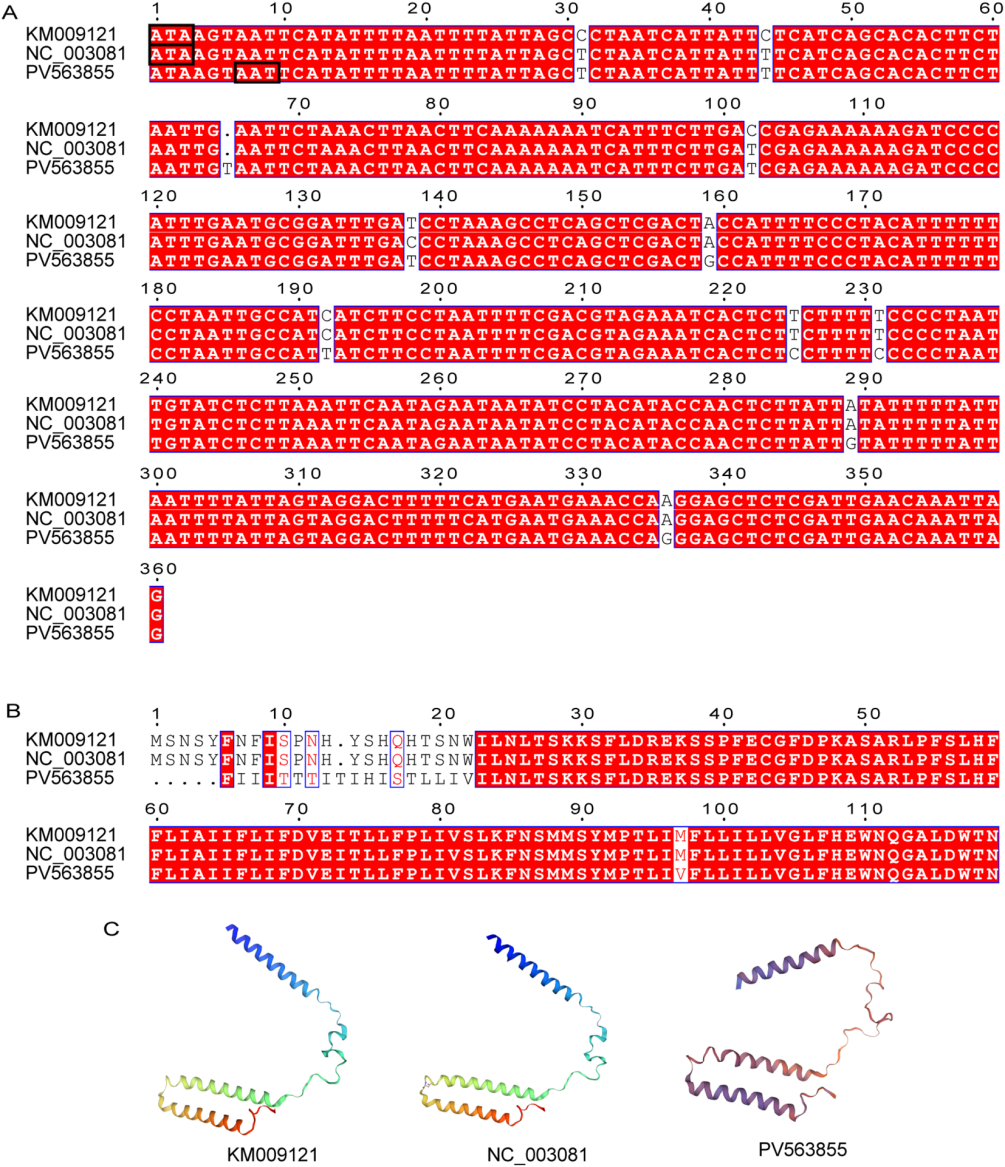
Alignment and sequence similarity analysis of *nd3* gene. **(A)** Nucleic acid sequences. Black box indicates the promoter; **(B)** Amino acid sequences; **(C)** Comparison of nd3 protein 3D structure prediction models.

In the present study, the *nad3* gene comprises 354 nucleotides encoding 117 amino acids, contrasting with previously reported 360-nucleotide sequences encoding 119 amino acids in other populations (5, 6). Sequence alignment revealed a thymine insertion at position 5,594 within the *nad3* locus (**Figure 3**). This single-nucleotide insertion induces a 7-bp frameshift (original initiation codon ATA at position 5,529 → revised ATT at position 5,536), resulting in complete divergence of the first 20 amino acid residues compared to published sequences. Crucially, this frameshift modifies the open reading frame (ORF), altering the protein’s tertiary structure at the N-terminus while preserving downstream structural domains (**Figure 3C**), suggesting potential compensatory mechanisms in mitochondrial translation.

### 3.3 tRNA and rRNA genes

All 22 tRNA genes were identified, with a combined length of 1,435 bp (range: 59–71 bp; **Table 3**). Notably, only *trnS1* (AGN) deviated structurally, lacking the dihydrouridine (DHU) arm characteristic of canonical cloverleaf folds (**Supplementary Figure S2**). The remaining 21 tRNAs exhibited standard cloverleaf secondary structures (**Supplementary Figure S2**). Gene boundaries were determined primarily by homology-based alignment using MITOS, with secondary-structure prediction (tRNAscan-SE) serving as validation. Eight tRNA genes were located on the minor strand, and fourteen on the major strand. The *rrnL* and *rrnS* genes measured 1,261 bp and 757 bp, respectively (**Table 3**).

### 3.4 Control region

The control region of *T. castaneum*, a highly variable segment critical for mitogenome replication and transcription initiation, spans 1,238 bp between trnI and rrnS (A+T content: 82.80%; AT-skew: -0.078; GC-skew: -0.001), with population-specific variations: 1,237 bp/82.30% A+T in the Jiangsu population and 1,239 bp/82.50% A+T in the California population (4). Sequence alignment revealed 26 single nucleotide variants (SNVs) between the reported mitochondrial genome control region and the Jiangsu population (6), 20 SNVs compared to the California population (5), including 7 shared SNVs (**Supplementary Figure S3**).

## 4 Discussion

The mitochondrial genome of *T. castaneum* from sauce-flavor Daqu, was sequenced as a circular molecule of 15,885 bp with a high A+T content (71.81%). It includes 13 protein-coding genes (PCGs), 22 tRNA genes, 2 rRNA genes, and a 1,238 bp control region (82.80% A+T). Key observations include unique initiation codons for *cox1* (CTG) and *nad1* (TTG), differing from previously reported populations, and a frameshift mutation in *nad3* caused by a thymine insertion at position 5,594, which altered the open reading frame (ORF) while preserving downstream structural domains. The control region showed minor variations in length (1,238 bp) and A+T content (82.80%) compared to the Jiangsu (1,237 bp; 82.30% A+T) and California (1,239 bp; 82.50% A+T) populations (5, 6).

The PCGs of the mitochondrial genome reported in this study differ from those of two previously reported populations, including single nucleotide variants (SNVs) and insertions/deletions (indels). SNVs caused nonsynonymous mutations in all genes except *atp6* and *cox3*. These mutations altered amino acid sequences, potentially modifying subunit secondary structures and interactions. We hypothesize that the observed SNVs may confer advantages in Daqu-adapted *T. castaneum*. This hypothesis requires validation through: (1) thermal tolerance tests comparing survival at 60-65 °C; (2) metabolic assays of detoxification enzymes; (3) fitness assessments tracking development and reproduction. A T-base insertion in the *nad3* gene changed its initiation site, resulting in distinct differences in the first 20 amino acids compared to the *nad3* genes of the Jiangsu (6) and California (5) populations, while subsequent sequences remained nearly identical. The *nad3* frameshift may signify adaptive remodeling under Daqu’s unique stresses, mirroring *T. castaneum* mitochondrial adaptations to environmental pressures. For instance, in cold-adapted *Laodelphax striatellus*, *cytB*/*nad5* mutations enhance ATP synthesis efficiency by 27% at 4°C despite reduced flight capacity (13); similarly, *Curculio chinensis* exhibits altitude-driven divergence in *atp6*/*8* genes (dN/dS=0.38) to optimize hypoxia tolerance (14). Analogously, the *nad3* frameshift mutation could reconfigure energy metabolism for Daqu’s high-temperature (60–65 °C) and acidic milieu, though functional validation remains essential.

The control region’s exceptional variability—demonstrated by length polymorphisms (1,238 bp vs. 1,237/1,239 bp in comparative strains) and 26 SNVs relative to the Jiangsu population (see Results 3.4)—aligns with its reduced selective constraints. This permits accumulation of neutral mutations through mechanisms like G-C tandem repeat expansion (15) and elevated mutation rates (16), paralleling patterns in Psittaciformes (17).

## 5 Conclusions

This study presents the first mitochondrial genome of *T. castaneum* adapted to sauce-flavor Daqu (15,885 bp; GenBank PV563855), revealing population-specific signatures including unique initiation codons (*cox1* CTG, *nad1* TTG), an *nad3* frameshift mutation, and control region polymorphisms. While methodological variations may influence genomic interpretations, these features suggest potential responses to Daqu-specific conditions like organic acids and high-temperature fermentation. Future investigations could examine functional implications through enzyme activity measurements and expanded geographical sampling across Chinese baijiu production regions, providing deeper insights into pest adaptation mechanisms relevant to fermented food industries.

## Data Availability

The datasets presented in this study can be found in online repositories. The names of the repository/repositories and accession number(s) can be found below: https://www.ncbi.nlm.nih.gov/genbank/, PV563855.

## Supplementary material

Supplementary Figure 1 The sequencing depth and coverage map for mitochondrial genomes.

Supplementary Figure 2 Predicted secondary structures of tRNA products encoded by tRNA genes. **(A)** *trnA* **(B)** *trnC* **(C)** *trnD* **(D)** *trnE* **(E)** *trnF* **(F)** *trnG* **(G)** *trnH* **(H)** *trnK* **(I)** *trnI* **(J)** *trnL1* **(K)** *trnL2* **(L)** *trnM* **(M)** *trnN* **(N)** *trnP* **(O)** *trnQ* **(P)** *trnR* **(Q)** *trnS1* **(R)** *trnS2* **(S)** *trnT* **(T)** *trnV* **(U)** *trnW* **(V)** *trnY*.

Supplementary Figure 3 Alignment and sequence similarity analysis of Control region.
