# Supplementary figures and images for: Complete mitochondrial genome of Tribolium castaneum (Coleoptera: Tenebrionidae) reared on sauce-flavor Daqu
Source: Front Insect Sci. 2025 Aug 29;5:1621855. doi: 10.3389/finsc.2025.1621855 (PMC12426077; doi:10.3389/finsc.2025.1621855)

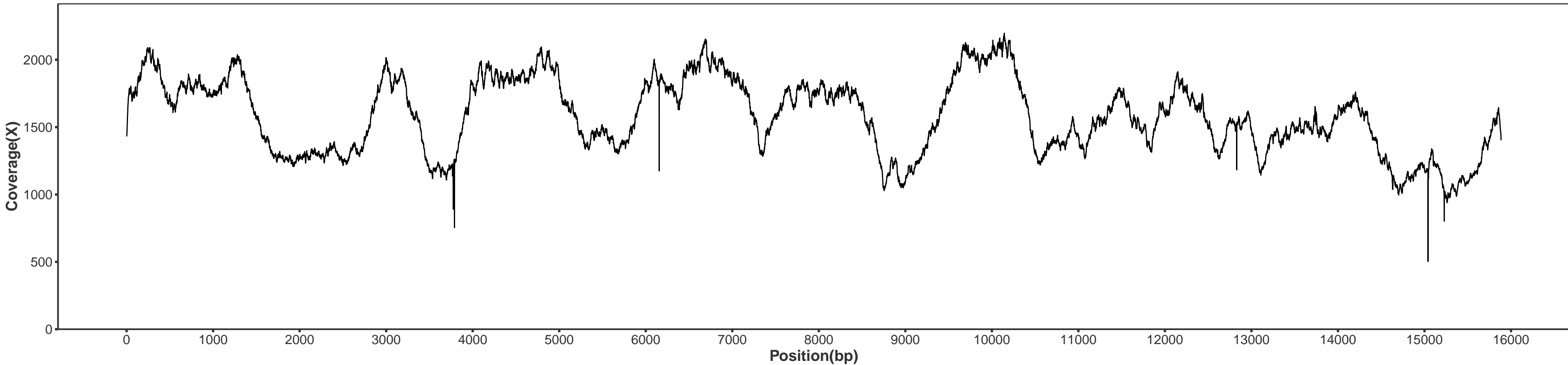

Supplement: Supplementary Figure 1 — The sequencing depth and coverage map for mitochondrial genomes. [file DataSheet1.pdf]

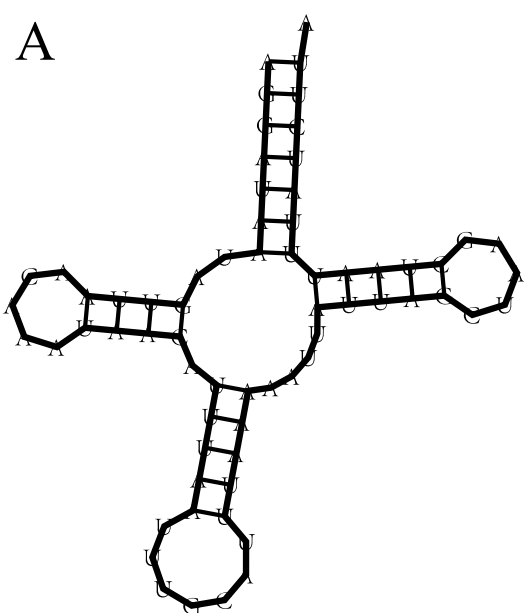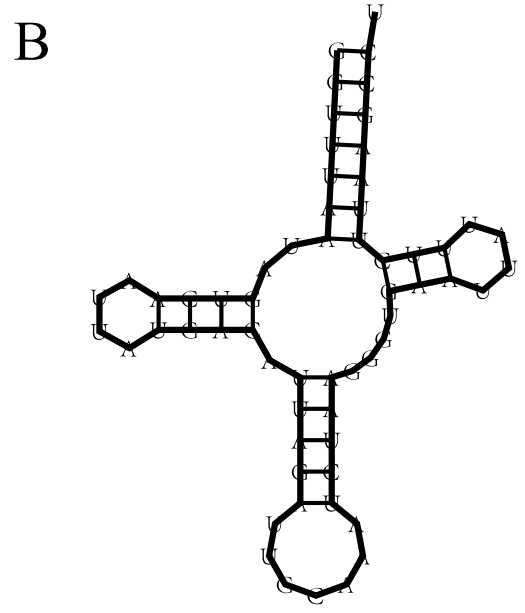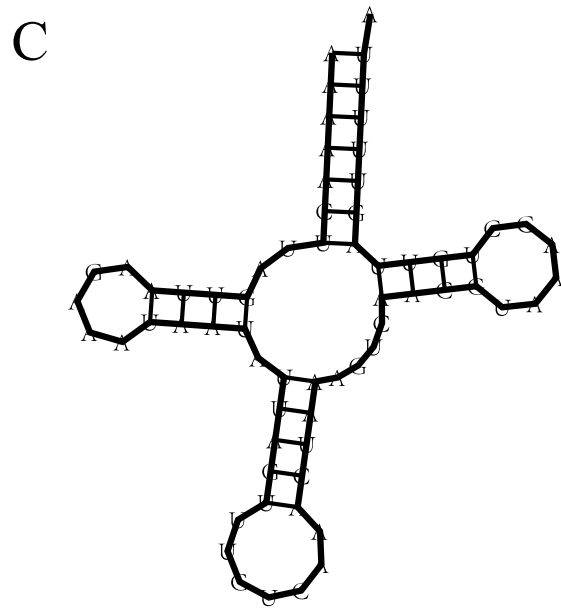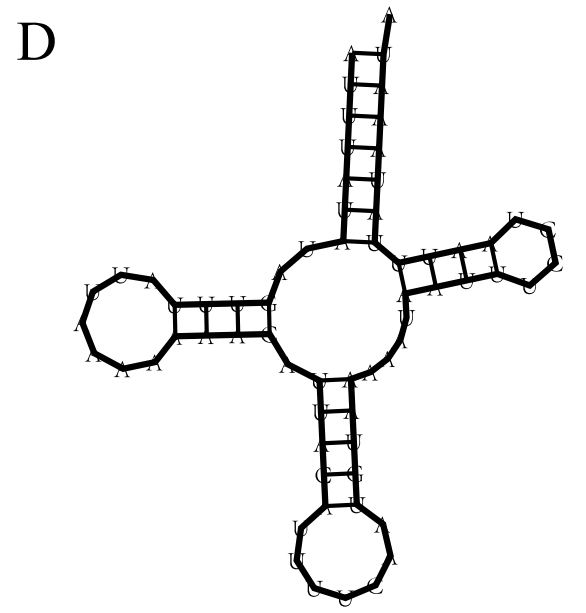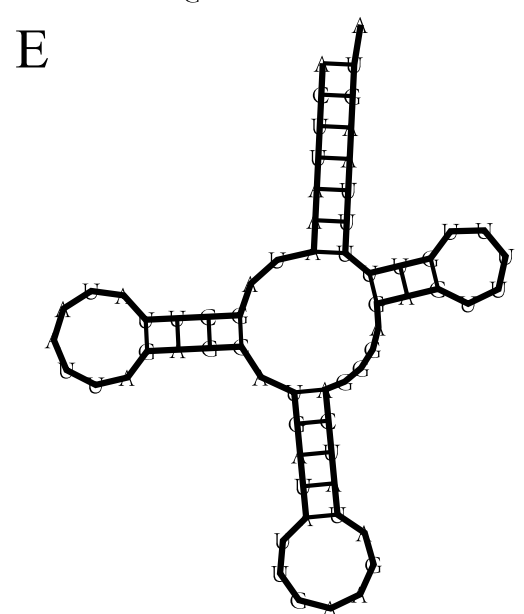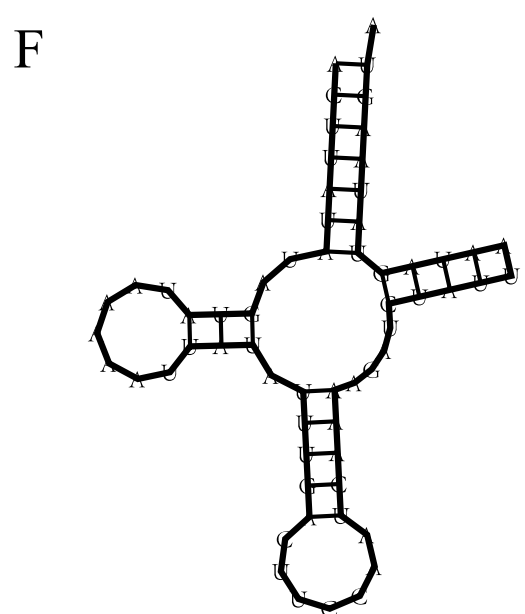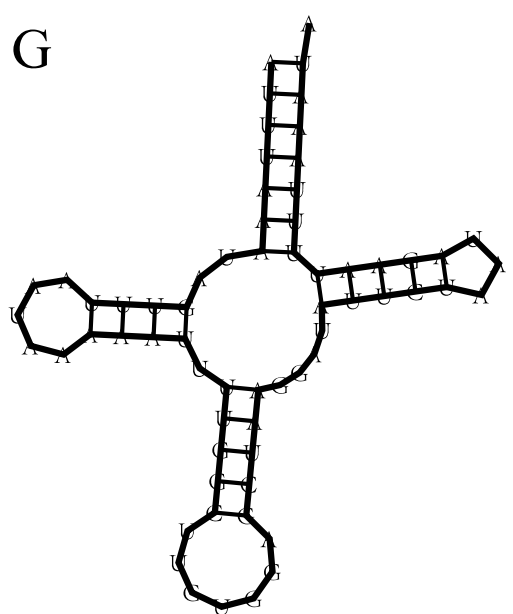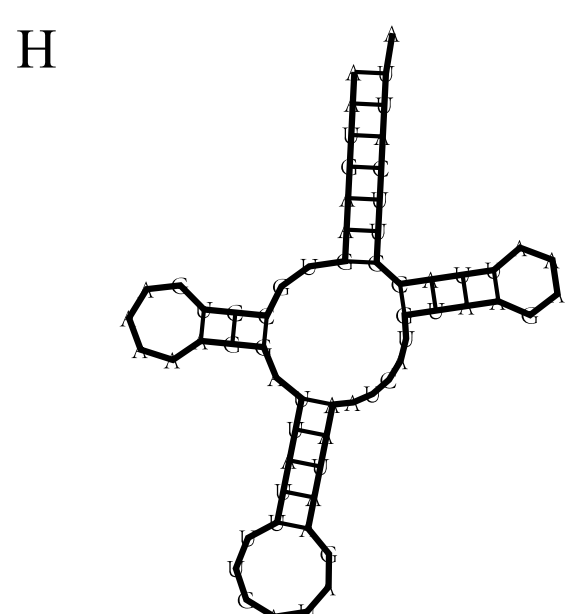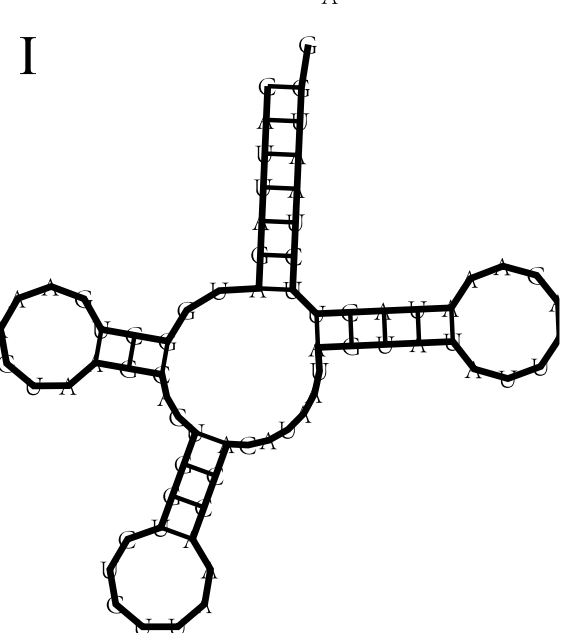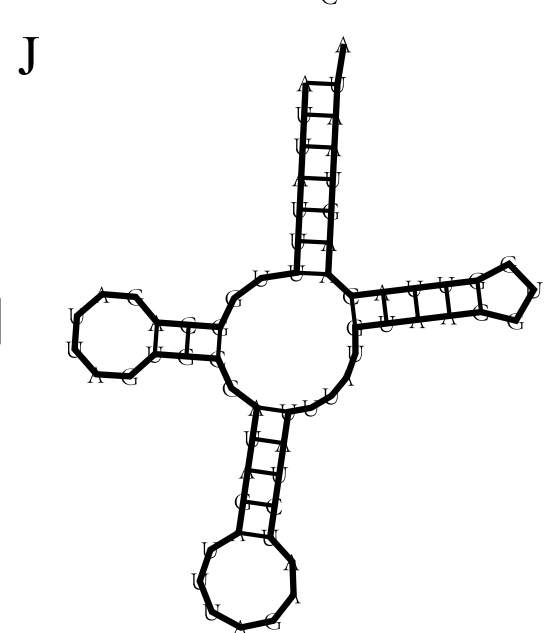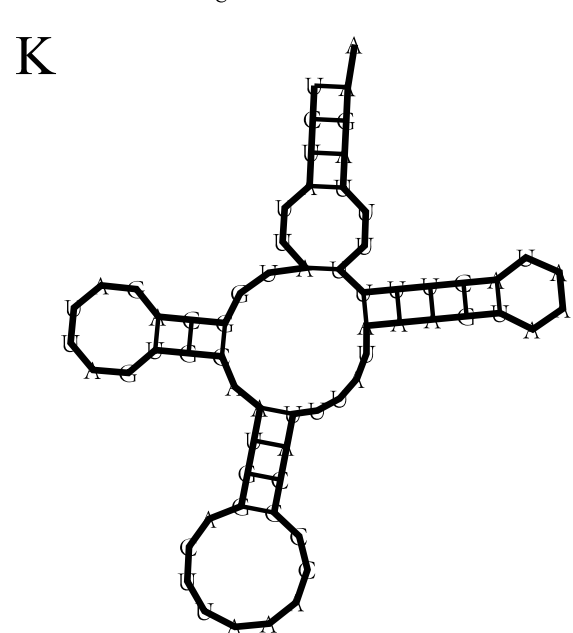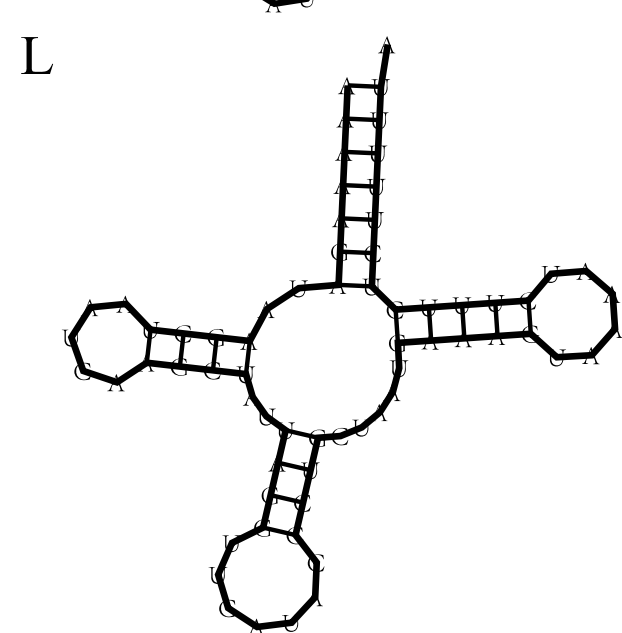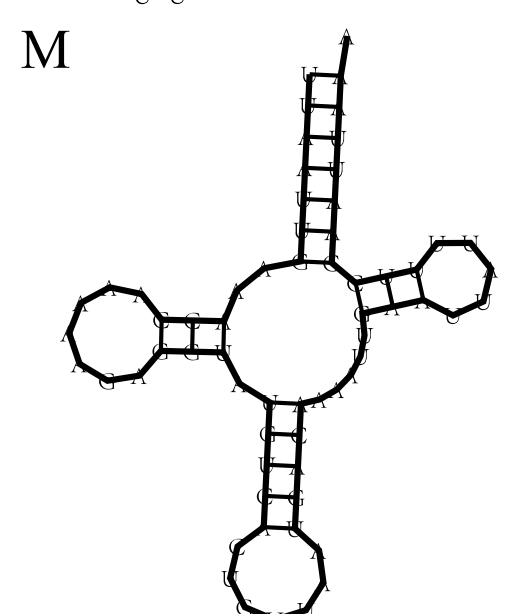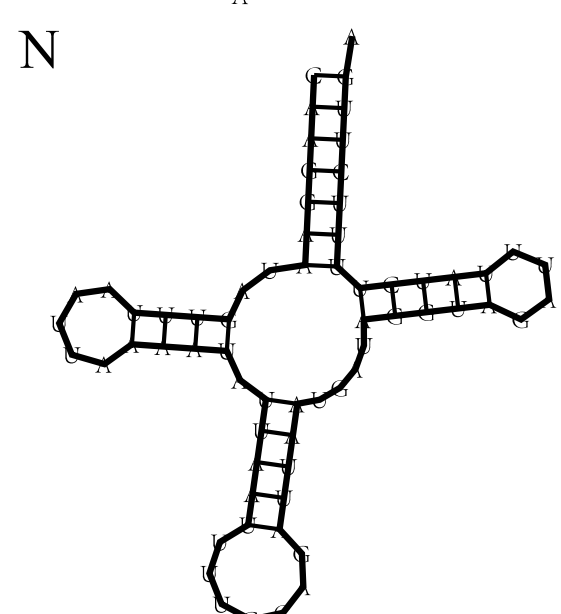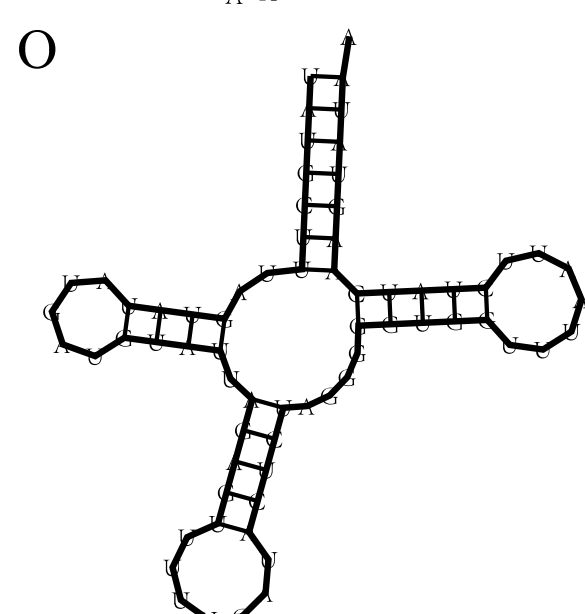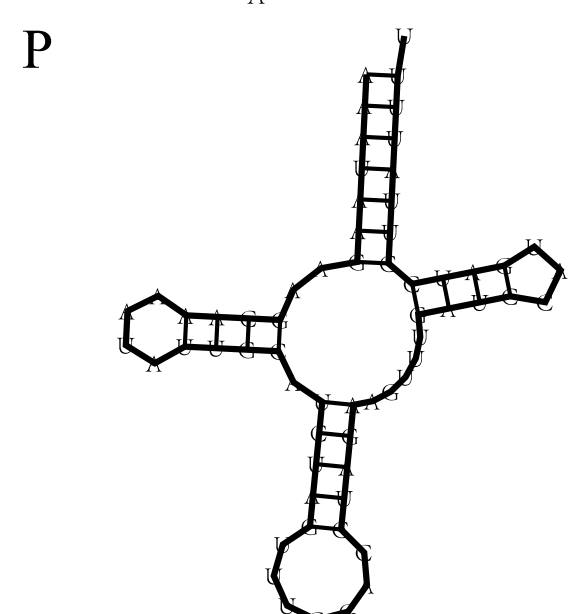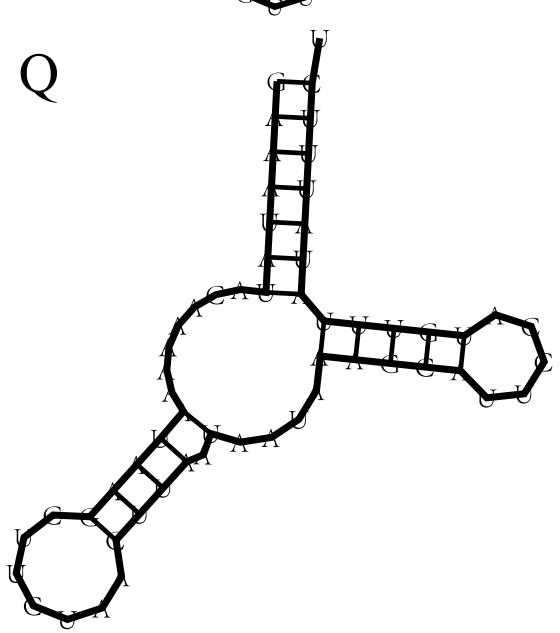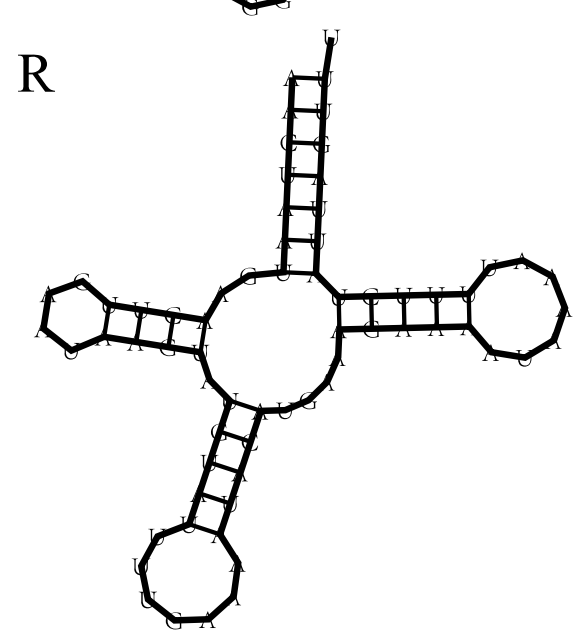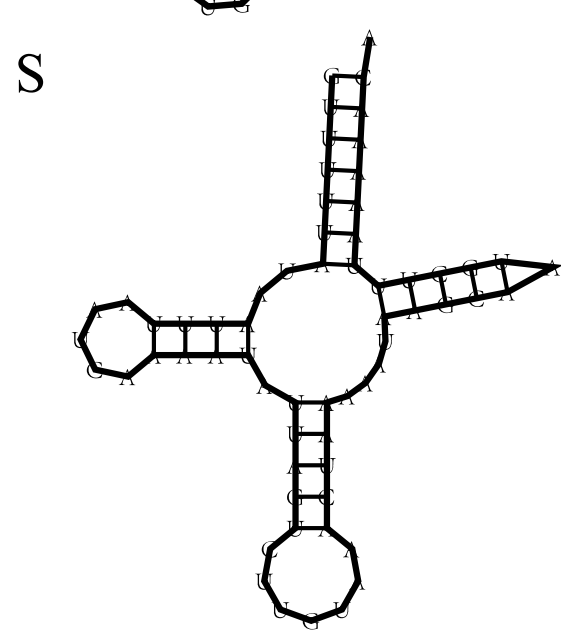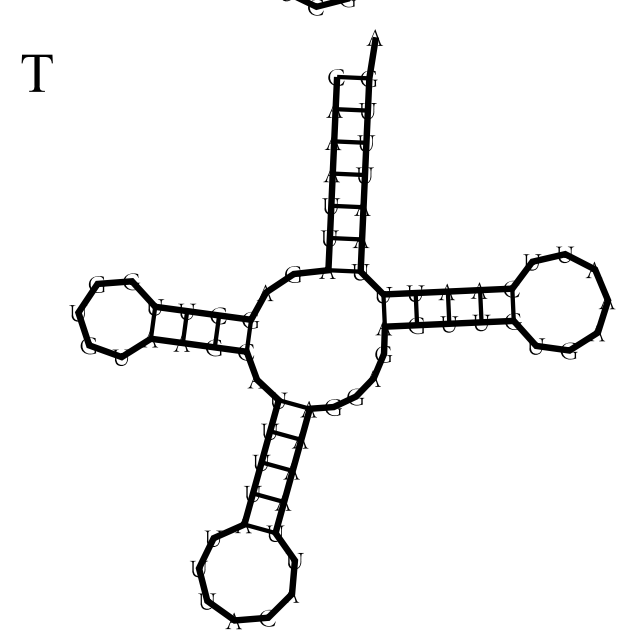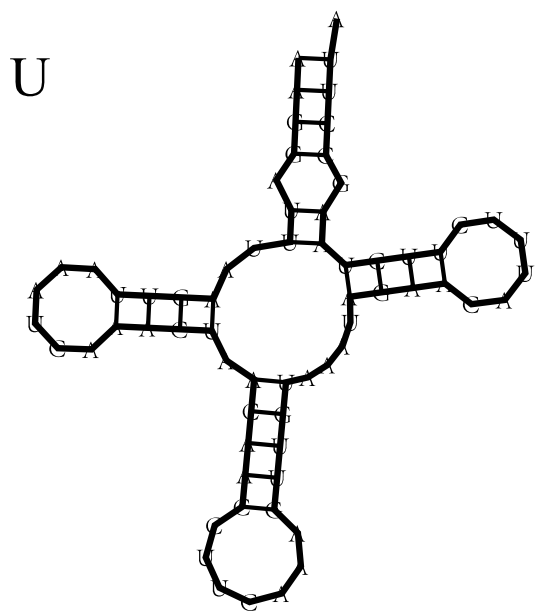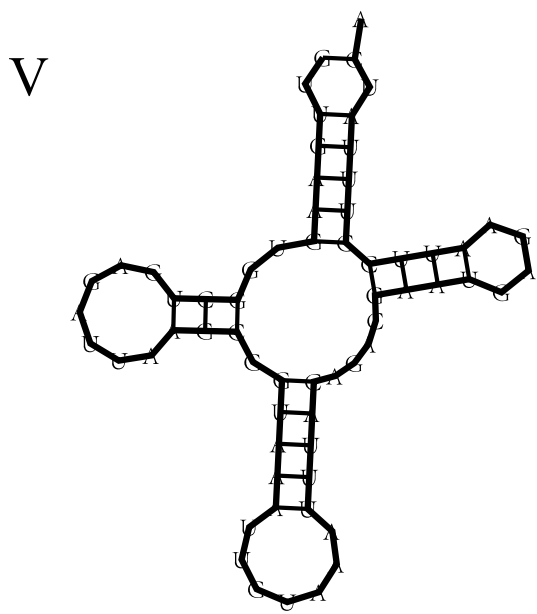

Supplement: Supplementary Figure 2 — Predicted secondary structures of tRNA products encoded by tRNA genes. (A) trnA (B) trnC (C) trnD (D) trnE (E) trnF (F) trnG (G) trnH (H) trnK (I) trnI (J) trnL1 (K) trnL2 (L) trnM (M) trnN (N) trnP (O) trnQ (P) trnR (Q) trnS1 (R) trnS2 (S) trnT (T) trnV (U) trnW (V) trnY. [file DataSheet2.pdf]
