# Supplementary material for: Complete mitochondrial genome of Tribolium castaneum (Coleoptera: Tenebrionidae) reared on sauce-flavor Daqu
Source: Front Insect Sci. 2025 Aug 29;5:1621855. doi: 10.3389/finsc.2025.1621855 (PMC12426077; doi:10.3389/finsc.2025.1621855)
Supplement: Supplementary Figure 3 — Alignment and sequence similarity analysis of Control region. [file DataSheet3.pdf]

```
                1        10        20        30        40        50        60
KM009121     TAACTCAAAGACAATCCTCCCTAAAAAAAGCCCGCTTGCTTAATTTTGCTCCCCTAAGCG
NC_003081    TAACTCAAAGACAATCCTCCCTAAAAAAAGCCCGCTTGCTTAATTTTGCTCCCCTAAGCG
PV563855     TAACTCAAAGACAATCCTCCCTAAAAAAAGCCCGCTTGCTTAATTTTGCTCCCCTAAGCG

                         70        80        90       100       110       120
KM009121     GGCTTAGAAAAATTTTTTCTAAAACATATAAATATTAATAGATTTTGCTTAATTCTAGAA
NC_003081    GGCCTAGAAAAATTTTTTCTAAAACATATTAATATTAATAGATTTTGCTTAATTCTAGAA
PV563855     GGCTTAGAAAAATTTTTTCTAAAACATATAAATATTAATAGATTTTGCTTAATTCTAGAA

                        130       140       150       160       170       180
KM009121     AAATTAAATTTATAGTAATCAACCCCTTTGCTTTAAAAATTTTCTTAATTATAAAGCATG
NC_003081    AAATTAAATTTATAATAATCAACCCCTTTGCTTTAAAAATTTTTTTAATTATAAAGCATG
PV563855     AAATTAAATTTATAGTAATCAACCCCTTTGCTTTAAAAATTTTCTTAATTATAAAGCATG

                        190       200       210       220       230       240
KM009121     ATTAAAACAATTTTTTATTCTAAAATAAATCTATTAATATTTAAGTAATCAAAAATTTTA
NC_003081    ATTAAAACAATTTTTTATTCTGAAATAAATCAATTAATATTTAAGTAATCAAAAATTTTA
PV563855     ATTAAAACAATTTTTTATTCTAAAATAAATCAATTAACATTTAAGTAATCAAAAATTCTA

                        250       260       270       280       290       300
KM009121     AATTAAACCTAAATACATTAATGTAAATTAAACCCCTCTTATTTTATTAATCCCCTAACT
NC_003081    AATTAAACCTAAATACATTAATGTAAATTAAACCCCTCTTATTTTATTAATCCCCTAACT
PV563855     AATTAAACTTAAATACATTAATATAAATTAATCCCCTCTTATTTTATTAATCCCCTAACT

                        310       320       330       340       350       360
KM009121     CAAAATAAAATAATATTAATTTAAATTAAAATATGAAAATTTATTTTTTATTACCTTTAT
NC_003081    CAAAATAAAATAATATTAATTTAAATTAAAATATGAAAATTTATTTTTTATTACCTTTAT
PV563855     CAAAATAAAATAATATTAATTTAAATTAAAATATGAAAATTTATTTTTTATTACCTTTAT

                        370       380       390       400       410
KM009121     GCTCAGAAAATAATTTCTCTGCAAATTTCAATT.AAAAAAATTTAGCTACAAC.TTTTTT
NC_003081    GCTCAGAAAATAATTTCTCTGCAAATTTCAATT.AAAAAAATTTAGCTACAACTTTTTTT
PV563855     GCTCAGAAAATAATTTCTCTGCAAATTTCAATTAAAAAAAATTTAGCTACAAC.TTTTTT

             420       430       440       450       460       470
KM009121     GTAAATTAAAACTTTTTTTAAAAAAAATTAATTTCTAACTCAATAAATTAATATTCACTT
NC_003081    GTAAATTAAAACTTTTTTAAAAAAAAATTAATTTCTAACTCAATAAATTAATATTCACTT
PV563855     GTAAGTTAAAACTTTTTTTAAAAAAAATTAATTTCTAACTCAATAAATTAATATTCACTT

             480       490       500       510       520       530
KM009121     ATTTTTTCAAATTTTACAGAAAAATAAATGTAATGAAATTTAAATTTCAACTAAATTCTT
NC_003081    ATTTTTTCAAATTTTACAGAAAAATAAATGTAACGAAATTTAAATTTTAACTAAATTCTT
PV563855     ATTTTTTCAAATTTTACAGAAAAATAAATGTAATGAAATTTAAATTTCAACTAAATTCTT

             540       550       560       570        580       590
KM009121     CAATTCAATGAAACTTGTGTATTAATGTAGGAAAGA.GGAAGTTTTTTTTTTTTTTCTAT
NC_003081    CAATTCAATGAAACTTGTGTATTAATGTAGGAAAGAGGGAAGTTTTTTTTTTTTTTCTAT
PV563855     CAATTCAATGAAACTTGTGTATTAATGTAGGAAAGA.GGAAGTTTTTTTTTTTTTTCTAT

              600       610       620       630       640       650
KM009121     TAAATATTTATGTATCATTATTAAATATATTAATTATTAATAATCGCTATTATATTTAAA
NC_003081    TAAATATTTATGTATCATTATTAAATATATTAATTATTAATAATCGCTATTATATTTAAA
PV563855     TAAATATTTATGTATCATTATTAAATATATTAATTATTAATAATCGCTATTATATTTAAA
```

```
            660       670       680       690       700       710
KM009121    TATTTAATCTTCGTTTAATGATTTATAAGTTTATATAGTTACATATTTATAAATAATTAT
NC_003081   TATTTAATCTCCGTTTAATGATTTATAAGTTTATATAGTTACATATTTATAAATAATTAT
PV563855    TATTTAATCTTCGTTTAATGATTTATAAGTTTATATAGTTACATATTTATAAATAATTAT

            720       730       740       750       760       770
KM009121    ATATATATAAATATTTAATTAATAAATAAAAGGAACTTAATACTAATCATATTCAACAAT
NC_003081   ATATATATAAATATTTAATTAATAAATAAAAGGAACTTAATACTAATCATATTCAACAAT
PV563855    ATATATATAAATATTTAATTAATAAATAAAAGGAACTTAATACTAATCATATTCAACAAT

            780       790       800       810       820       830
KM009121    AATCGGATTTTCCTTTCACTTAGCAAGCGATGGTTAATCCTGGGATAATTATAGTTATAT
NC_003081   AATCGGATTTTCCTTTCACTTAGCAAGCGATGGTTAATCCTGGGATAATTATAGTTATAT
PV563855    AATCGGATTTTCCTTTCACTTAGCAAGCGATGGTTAATCCTGGGATAATTATAGTTATAT

            840       850       860       870       880       890
KM009121    AAGAATATTAAATTCATTAAATACGTTATATATTCATATAAAATTATTAATTATATATAA
NC_003081   AAGAATATTAAATTCATTAAATACGTTATATATTCATATAAAATTATTAATTATATATAA
PV563855    AAGAATATTAAATTCATTAAATACGTTATATATTCATATAAAATTATTAATTATATATAA

            900       910       920       930       940       950
KM009121    CTTTTATTAATTATATAAAAATTATATATAAAGCTAATTATTACATTCTAATATATAATT
NC_003081   CTTTTATTAATTATATAAAAATTATATATAAAGCTAATTATTACATTCTAATATATAATT
PV563855    CTTTTATTAATTATATAAAAATTATATATAAAGCTAATTATTACATTCTAATATATAATT

            960       970       980       990       1000      1010
KM009121    ATATTATATATAAATCAATGCTTATCATATATATAATTATATATCCCATGATAATTAAAT
NC_003081   ATATTATATATAAATCAATGCTTATCATATATATAATTATATATTCCATGATAATTAAAT
PV563855    ATATTATATATAAATCAATGCTTATCATATATATAATTATATATTCCATGATAATTAAAT

            1020      1030      1040      1050      1060      1070
KM009121    AAGTTTTAATGTAGGTACCTTTTTTTATGAAATTTTTAGAGAAAAATTGACATCTTTAA
NC_003081   AAGTTTTAATGTAAGCACCTTTTCTTATGAAATTTTTAGAGAAAAATTGTCTTCTTTGA
PV563855    AAGTTTTAATGTAAGCACCTTTTTTTATGAAATTTTTAGAGAAAAATTGTCTTCTTTGA

            1080      1090      1100      1110      1120      1130
KM009121    CATTTGCTAATGTTAACTTAAATTGAGGAAAAATTGATGATGCAAAAAAATGCAATTTTC
NC_003081   CATTTACTAATGTTAACTTAAATTGAGGAAAAATTGATGATGCAAAAAAATGCAATTTTC
PV563855    CATTTACTAATGTTAACTTAAATTGAGGAAAAATTGATGATGCAAAAAAATGCAATTTTC

            1140      1150      1160      1170      1180      1190
KM009121    GTGAATTTTCTAAAAATTAGACTTAAAAATATTAACTAAACCAAACTTCATTGAATTAGG
NC_003081   GTGAATTTTCCAAAAATTAGACTTAAAAATATTAACTAAACCTAAATTAATTAAATTAAA
PV563855    GTGAATTTTCTAAAAATTAGACTTAAAAATATTAACTAAACCAAAATTAATTAAATTAAA

            1200      1210      1220      1230
KM009121    CTTATGAAAGTTGCGAAATTTAAATTACAAATAAAGTTTT
NC_003081   TTCAAGAAAGTTGTGAAACTTAAATTACAAATAAAGTTTT
PV563855    TTCAAGAAAGTTGTGAAATTTAAATTACAAATAAAGTTTT
```
